# Exploring the Impact of Personalized Nutritional Approaches on Metabolism and Immunity: A Systematic Review of Various Nutrients and Dietary Patterns

**DOI:** 10.7759/cureus.58553

**Published:** 2024-04-18

**Authors:** Christian N Paulsingh, Muhammad Faisal Riaz, Gourav Garg, Lotanna Umeano, Sadaf Iftikhar, Sarah F Alhaddad, Pousette Hamid

**Affiliations:** 1 Pathology, St. George's University School of Medicine, St. George's, GRD; 2 Internal Medicine, California Institute of Behavioral Neurosciences & Psychology, Fairfield, USA; 3 Internal Medicine, Rawalpindi Medical University, Rawalpindi, PAK; 4 Orthopedics, Kings Mill Hospital, Sutton-in-Ashfield, GBR; 5 Pediatrics, California Institute of Behavioral Neurosciences & Psychology, Fairfield, USA; 6 Neurology, California Institute of Behavioral Neurosciences & Psychology, Fairfield, USA

**Keywords:** nutrition, diet, immunometabolism, immune function, immunity, micronutrients, macronutrients, nutrition and metabolism, digestion

## Abstract

The systematic review investigates the impact of different nutrients and dietary patterns on metabolism and immunity to answer the research question: "Can personalized nutritional approaches boost immunity?" The importance of diet in supporting the immune system has come to light in today's environment, where a strong immune system is crucial for protection against infectious illnesses, as highlighted by the COVID-19 pandemic. This systematic review adhered to the guidelines outlined in the Preferred Reporting Items for Systematic Reviews and Meta-Analyses (PRISMA) 2020. Four databases were screened for relevant data published in 2022-2023: PubMed, PubMed Central (PMC), MEDLINE, and Cochrane Library. Inclusion and exclusion criteria were utilized, and 13 papers were finalized after screening and employing the quality appraisal tool Cochrane Bias assessment for randomized controlled trials (RCT). Personalized nutrition can strengthen immunity and enhance overall health by adjusting dietary recommendations and following a person's genetic makeup, lifestyle, and health state. An adequate supply of vitamins, minerals, proteins, and fatty acids as well as an optimum caloric intake are essential for immune health, and individual requirements can vary significantly due to genetic factors, lifestyle choices, and underlying health conditions. Personalized nutrition considers these factors, enabling tailored dietary recommendations to address specific nutrient needs and optimize nutrient intake, leading to better health outcomes. The review concludes that personalized nutrition is more effective than a one-size-fits-all approach in boosting immunity, and its potential impact on health and immune function is highly important.

## Introduction and background

According to the World Health Organization (WHO), malnutrition often refers to wasting, stunting, and being underweight, reflecting imbalances such as insufficient vitamins and minerals. Malnutrition also covers terms such as being overweight or obese and is associated with non-communicable illnesses linked to food [1]. In 2021, the WHO estimated that 1.9 billion adults are overweight or obese, while 462 million are underweight [1].

The importance of diet in supporting the immune system has come to light in today's dynamic environment, in which health and well-being are top priorities. In addition to other health issues, the COVID-19 pandemic has highlighted the importance of a strong immune system to protect the body against infectious illnesses [2,3]. Individualized dietary techniques are effective for improving an individual’s immune function. Personalized nutrition can strengthen immunity and enhance overall health by adjusting dietary recommendations following a person's particular genetic makeup, lifestyle, and health state [4]. The body's defense against hazardous pathogens such as bacteria, viruses, and fungi is provided by the immune system and a vast network of cells, tissues, and organs [4,5]. It consists of innate and adaptive immune responses, with the former providing immediate, nonspecific protection and the latter offering a more targeted, long-term defense mechanism. The efficacy of the immune system is influenced by several variables including heredity, age, way of life, and diet [5-7].

The modulation of immune function is significantly affected by nutrition. A robust immune system must be developed and maintained, which requires an adequate intake of vitamins, minerals, proteins, and fatty acids. Examples of vitamins that are known to boost immunological function include vitamin C and vitamin D. Collagen, a protein that aids in wound healing and creates a barrier against infections, is produced only when vitamin C is present in the body [8]. In contrast, vitamin D assists in regulating the immune system and lessens inflammation [9].

Minerals such as zinc and selenium are vital for immune health. Zinc is involved in the development and function of immune cells, and its deficiency can impair the immune response [10]. Selenium acts as an antioxidant that protects immune cells from oxidative damage [11]. These nutrients, among others, are essential for immune cell proliferation, cytokine production, and antibody responses [8-11]. Moreover, proteins are essential for the synthesis of antibodies and immune cells, which are crucial components of the immune system. Amino acids, which are the building blocks of proteins, play a role in the production of antibodies and other immune molecules. Therefore, a balanced intake of high-quality proteins is essential for optimal immune function [12].

Although the importance of essential nutrients for immune health is well established, the concept of personalized nutrition recognizes that individual requirements can vary significantly. Genetic factors, lifestyle choices, and underlying health conditions influence an individual's nutritional needs and metabolism. Genetic variations can affect how the body processes and utilizes nutrients. For example, some individuals may have genetic variations that affect their ability to efficiently metabolize certain vitamins or minerals [13]. Personalized nutrition considers these genetic factors, enabling tailored dietary recommendations to address specific nutrient needs. Lifestyle choices such as exercise habits, stress levels, and sleep patterns also influence immune function. A personalized nutrition approach considers these factors when crafting dietary recommendations. For instance, individuals with high stress levels may benefit from foods rich in stress-reducing nutrients such as magnesium and B vitamins [14,15]. Furthermore, underlying health conditions can affect nutrient absorption and utilization. Individuals with conditions such as celiac disease or lactose intolerance may have specific dietary restrictions that require careful consideration in personalized nutrition plans. By addressing these unique factors, personalized nutrition can optimize nutrient intake and improve immune function [16].

Personalized nutrition is demonstrably more effective than a one-size-fits-all in boosting immunity as we discovered when we examined its potential. The topic of personalized nutrition and its potential impact on health and immune function is highly important as it has the potential to improve health outcomes, prevent chronic diseases, and support immune function in individuals with specific needs or health conditions. Personalized nutrition may address genetic variations, lifestyle factors, and health conditions that influence nutrient requirements and metabolism, leading to better health outcomes. Our findings suggest that personalized nutrition may be particularly beneficial for individuals with compromised cardiometabolic profiles as favorable effects on cardiometabolic health were noted in these individuals. This may include individuals with conditions such as obesity, type 2 diabetes, and cardiovascular diseases. Various nutrients and dietary patterns should be investigated to understand their effects on metabolism and immunity. The study aims to shed light on the exciting prospects of enhancing immunity through customized dietary recommendations based on an analysis of the existing literature and emerging research on personalized nutrition.

## Review

Methods

The Preferred Reporting Items for Systematic Reviews and Meta-Analyses (PRISMA) 2020 checklist was used to search for the relevant literature [17].

Search Sources and Search Strategy

Four databases were used in this study: PubMed, MEDLINE, PubMed Central (PMC), and Cochrane Library. In the PubMed database, a Medical Subject Heading (MeSH) concept was established with the use of three key terms and combined with the Boolean “AND.” For the first concept term “nutrition,” we used similar concept identification words such as diet, food, nourishment, menu, nutriment, sustenance, micronutrients, and macronutrients. These were separated by the Boolean “OR” and incorporated into the MeSH search. The same was done for the other key concept “immunity” with synonyms such as autoimmune, immune, protection, safe, invulnerable, and unsusceptible. The last concept “metabolism” was merged with terms such as basic metabolic rate, digestion, consumption, ingestion, catabolism, anabolism, glycolysis, glycogenolysis, gluconeogenesis, glycogenesis, tricyclic acid cycle, fatty acid oxidation, fatty acid synthesis, cholesterol synthesis, ketogenesis, purine synthesis, pyrimidine synthesis, and hexose monophosphate shunt. The MeSH strategies were compiled to produce a completed search term for the MeSH database. Table 1 displays the details of the search strategies that were employed in this systematic review.

**Table 1:** Elements of the search strategy used in this systematic review

Database	Search strategy	Filters applied	Results
PubMed, PubMed Central (PMC), MEDLINE	Nutrition OR diet OR food OR nourishment OR menu OR nutriment OR sustenance OR micronutrients OR macronutrients OR (("Nutritional Status/drug effects"[Majr] OR "Nutritional Status/ethnology"[Majr] OR "Nutritional Status/genetics"[Majr] OR "Nutritional Status/immunology"[Majr] OR "Nutritional Status/physiology"[Majr])) OR ("Nutritional Status/drug effects"[Majr:NoExp] OR "Nutritional Status/ethnology"[Majr:NoExp] OR "Nutritional Status/genetics"[Majr:NoExp] OR "Nutritional Status/immunology"[Majr:NoExp] OR "Nutritional Status/physiology"[Majr:NoExp]) AND Immunity OR autoimmune OR immune OR protection OR safe OR invulnerable OR unsusceptible OR (("Immunity/drug effects"[Majr] OR "Immunity/genetics"[Majr] OR "Immunity/immunology"[Majr] OR "Immunity/physiology"[Majr])) OR ("Immunity/drug effects"[Majr:NoExp] OR "Immunity/genetics"[Majr:NoExp] OR "Immunity/immunology"[Majr:NoExp] OR "Immunity/physiology"[Majr:NoExp]) AND Metabolism OR basic metabolic rate OR digestion OR consumption OR ingestion OR catabolism OR anabolism OR glycolysis OR glycogenolysis OR gluconeogenesis OR glycogenesis OR tricyclic acid cycle OR fatty acid oxidation OR fatty acid synthesis OR cholesterol synthesis OR ketogenesis OR purine synthesis OR pyrimidine synthesis OR hexose monophosphate shunt OR (("Metabolism/drug effects"[Majr] OR "Metabolism/genetics"[Majr] OR "Metabolism/immunology"[Majr] OR "Metabolism/physiology"[Majr])) OR ("Metabolism/drug effects"[Majr:NoExp] OR "Metabolism/genetics"[Majr:NoExp] OR "Metabolism/immunology"[Majr:NoExp] OR "Metabolism/physiology"[Majr:NoExp])	Free full text available, English language, published in the last 1 year, randomized controlled trials (RCT) only, adult 19+ years, humans	786
Cochrane	(Nutrition OR diet) AND (Immunity OR autoimmune OR immune) AND (Metabolism OR basic metabolic rate)	English language, published in the last 1 year	62

The database search was completed on September 19, 2023, and all our final selected papers were identified. Our inclusion and exclusion criteria were used to reduce the number of articles due to the large number of papers found. After screening the articles based on titles and abstracts, we finalized our selection by checking data quality. A systematic review of the 13 studies was conducted.

Inclusion Criteria

We narrowed our selection to articles in English published within the year 2022-2023 that focused on adults aged ≥ 18 years. Studies that focused only on human participants and had free open access were accepted.

Exclusion Criteria

Our exclusion criteria were articles in languages other than English and papers published outside the period of 2022-2023. Gray literature and studies other than randomized controlled trials (RCT) were excluded, and animal and/or mixed-species studies were rejected.

Results

Initially, 848 articles were found in the PubMed, MEDLINE, PMC, and Cochrane Library databases combined. After identifying the records, one duplicate paper was removed. These 847 articles that remained were screened, and 827 were removed based on their titles and abstracts. Following the screening, based on the eligibility criteria of being published from 2022 to 2023 with a focus on the adult, human population being ≥ 18 years and papers that were published in English, 20 papers were assessed for quality using the quality appraisal tool namely Cochrane Risk of Bias Tool. This was used primarily on the basis that 16 papers were RCT, three were randomized crossover trials, and one was a pilot study. Papers were assessed based on whether there was a high, low, or unclear risk of bias for each criterion. Seven papers were removed because they did not meet the standards of quality appraisal.

**Figure 1:**
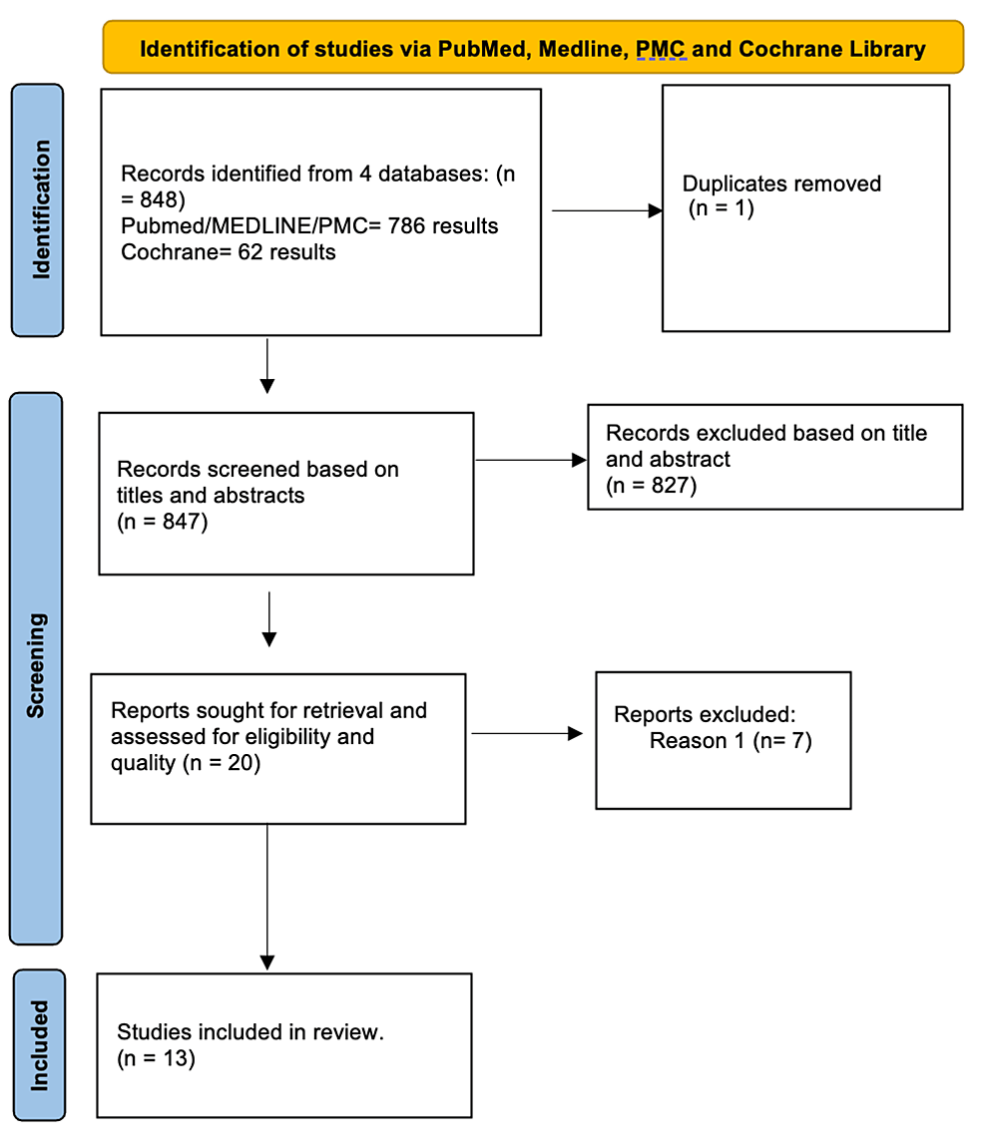
PRISMA flow diagram demonstrating the selection process of the included articles Reason 1: The paper had a low quality of study as assessed by a quality assessment tool. Source: Page et al. [17]. PRISMA: Preferred Reporting Items for Systematic Review and Meta-Analyses.

The PRISMA flowchart in Figure 1 shows the process by which the final studies were selected.

Table 2 displays the quality appraisal tool results of the final studies.

**Table 2:** Quality appraisal tool results of the studies included in this systematic review

Author and year of publication	Sequence generation	Allocation concealment	Blinding participants/ personnel	Blinding outcome assessors	Incomplete outcome data	Selective outcome reporting	Other sources of bias
Abuawad et al. [18] 2023	Low	Low	Low	Low	Low	Low	Unclear
Anderson et al. [19] 2023	Low	Low	Low	Low	Low	Unclear	Unclear
Arbizu et al. [20] 2023	Low	Low	Unclear	Unclear	Low	Low	Low
Basu et al. [21] 2023	Low	Low	Unclear	Low	Low	Unclear	Low
Ben-Yacov et al. [22] 2023	Low	Low	Low	Low	Low	Unclear	Low
De Guiseppe et al. [23] 2023	Low	Low	Low	Low	Unclear	Low	Low
Farsi et al. [24] 2023	Low	Low	Low	Low	Low	Low	Low
Fraschetti et al. [25] 2022	Low	Low	Unclear	Unclear	Low	Low	Low
Galena et al. [26] 2022	Low	Unclear	Low	Low	Low	Low	Low
Jung et al. [27] 2023	Low	Unclear	Unclear	Low	Low	Low	Low
Le Sayec et al. [28] 2022	Low	Low	Low	Low	Low	Unclear	Low
Tingö et al. [29] 2022	Low	Low	Low	Low	Low	Low	Low
van den Belt et al. [30] 2022	Low	Low	Low	Low	Low	High	Low

Table 3 represents a detailed description of the final studies' purpose, the outcomes that were measured, the location of the population studied, the number of participants and their respective age ranges, and the conclusion.

**Table 3:** A detailed description of the selected studies PMI: Primary methylation index; MMA: Monomethyl arsenic; bMMAs: Blood MMAs; InAs: Inorganic arsenic species; SMI: Secondary methylation index; DMAs: Dimethyl arsenic species; bDMAs: Blood DMAs; IL: Interleukin; TNF: Tumor necrosis factor; TGF: Transforming growth factor.

Author and year of publication	Study design	Purpose of study	Outcome measured	Number and age of participants	Location	Conclusion
Abuawad et al. [18] 2023	Randomized controlled trial	To determine the impact of folic acid and creatine supplementation on arsenic methylation indices and metabolite concentrations in a Bangladeshi population	The study measured outcomes encompass the levels of arsenic metabolites, along with the (PMI: MMAs/InAs) and the secondary methylation index (SMI: dimethyl arsenic species (DMAs)/MMAs) in the blood of Bangladeshi adults, who exhibit a diverse range of folate status.	622 participants ranged from 24 to 55 years, with a geometric mean (GM) of 37.5 ± 1.2 years	Bangladesh	Supplementing with folic acid resulted in decreased levels of blood MMAs(bMMAs) and increased levels of blood DMAs (bDMAs) in a group primarily consisting of adults with adequate folate levels. Conversely, creatine supplementation led to a reduction in bMMAs.
Anderson et al. [19] 2023	Randomized crossover trial	To assess how following various egg-based diets affected the daily nutrient intake as a whole and compare the changes in outcome measures during the egg white and whole egg phases in female participants, distinguishing between those who used combined oral contraceptives (COC) and those who did not.	The study's outcome measures encompassed alterations in clinical metabolic and hematological factors, such as changes in body mass index, body weight, body fat composition, and the overall daily nutrient intake from the diet.	29 participants with an age range of 18 to 35 years	United States of America	Consuming whole eggs resulted in more significant overall enhancements in micronutrient diet quality, choline levels, and high-density lipoprotein (HDL) and hematologic parameters, with minimal, albeit potentially less detrimental, effects on insulin resistance markers when compared to egg whites.
Arbizu et al. [20] 2023	Randomized controlled trial	The study aims to explore how supplementing with dark sweet cherries affects blood pressure and inflammation in adults who are obese, with a focus on potential health benefits.	Outcomes measured in this study include anthropometric measurements such as body weight, height, body mass index BMI, waist, and hip circumference as well as physiological biomarkers such as temperature, systolic and diastolic blood pressure, heart rate, and oxygen saturation. Blood samples were also collected to analyze lipid profiles, glucose levels, and liver enzymes. The study also assessed dietary intake and adherence to the Dietary Guidelines for Americans (DGA) using the Healthy Eating Index (HEI).	60 participants aged 18 years and older	United States of America	Incorporating dark sweet cherry supplements into the diets of obese adults resulted in lowered blood pressure and reduced levels of the pro-inflammatory substance interferon-gamma (IFNγ). Interestingly, these improvements did not impact factors such as lipid profile, glucose levels, or liver enzymes.
Basu et al. [21] 2023	Randomized controlled trial	To examine the impacts of including strawberries in the dietary habits of adults displaying signs of metabolic syndrome on the levels of serum metabolites associated with cardiometabolic risks.	The research conducted extensive assessments of serum metabolites, encompassing both focused and untargeted metabolites, at various intervals during the study. Among the targeted metabolites were serum branched-chain amino acids (BCAAs), while untargeted metabolites encompassed serum phosphate, benzoic acid, and hydroxyphenyl propionic acid.	33 participants enrolled aged 53 ± 13 years	United States of America	Incorporating whole strawberries into one's regular diet could offer a valuable and practical approach to enhance the cardiometabolic well-being of adults. This research reinforces the potential of strawberry supplementation in positively influencing the serum metabolic profiles linked to lower risks of insulin resistance, diabetes, and endothelial dysfunction among adults exhibiting signs of metabolic syndrome.
Ben-Yacov et al. [22] 2023	Randomized clinical trial	The research aims to explore how the gut microbiome is involved in the connection between diet and human well-being and to evaluate the impacts of tailored post-meal diets on cardiometabolic indicators among individuals at risk of developing diabetes.	The study assessed a range of cardiometabolic indicators, including measurements of glucose levels, insulin resistance, lipid levels, and body weight, among other factors. Additionally, the research incorporated a detailed examination of the dietary and microbiome composition changes over six months in participants who successfully completed the intervention.	800 participants aged 18 to 65 years	Israel	The study's findings indicated that tailoring nutrition plans to an individual's gut microbiome can enhance how the body responds to glucose after meals and positively impact various cardiometabolic indicators. Moreover, the research highlighted that the gut microbiome plays a role in influencing how dietary interventions affect these markers.
De Guiseppe et al. [23] 2023	Randomized controlled trial	The study aimed to explore the impact of crackers enriched with Camelina oil on the levels of omega-3 in the bloodstream as well as on inflammatory markers and serum lipid profiles in older adults aged 65 years and above. It sought to ascertain whether incorporating Camelina oil-enriched crackers into the diet could lead to improvement in these health parameters within this age group.	1. Primary outcome: Serum omega-3 fatty acid concentrations, including eicosapentaenoic acid (EPA), docosahexaenoic acid (DHA), linoleic acid (LA), and arachidonic acid (ARA) levels. 2. Secondary outcomes: Inflammatory status parameters, including pro-inflammatory cytokines (IL-18 and TNF-alpha), anti-inflammatory cytokines (TGF-beta 1 and TGF-beta 2), and C-reactive protein (CRP) levels. Serum lipid panel parameters, including total cholesterol (TC), high-density lipoproteins cholesterol (HDL), low-density lipoproteins cholesterol (LDL), and triglycerides (TG) concentrations.	66 participants aged 64-80 years, with a mean age of 70.6 years	Italy	The study's findings indicated that incorporating Camelina oil-enriched crackers into the diet led to a substantial rise in omega-3 levels in the bloodstream as well as improvements in inflammatory markers and the serum lipid profile among older adults. This suggests that Camelina oil might serve as a sustainable and wholesome substitute for conventional oils, offering potential benefits for enhancing the nutritional status and well-being of older individuals.
Farsi et al. [24] 2023	Randomized controlled trial	The aim of this study was to examine how replacing meat with mycoprotein impacts the composition of the gut microbiota, indicators of gut health, and genotoxicity in healthy adults.	In this study, the primary focus was on assessing fecal genotoxicity and genotoxins as the main objectives, with secondary objectives including the examination of alterations in the composition and activity of the gut microbiome. Additionally, the research investigated the presence of particular gut microorganisms and the excretion of short-chain fatty acids.	20 participants aged 18–50 years	North-East of England and the United Kingdom	The findings indicate that substituting meat with mycoprotein could potentially offer advantages in terms of gut health and genotoxicity.
Fraschetti et al. [25] 2022	Randomized crossover trial	To provide insights into the possible advantages of consuming milk to alleviate post-exercise inflammation.	The outcomes measured in this study were the concentrations of inflammatory biomarkers (IL-6, TNF-α, and IL-10) in the blood of untrained females after consuming either skim milk or an isoenergetic, isovolumetric carbohydrate drink following high-intensity/impact exercise.	13 participants aged 20.3 ± 2.3 years	Canada	The research revealed that after engaging in high-intensity or high-impact exercise, individuals who consumed skim milk exhibited reduced levels of the inflammatory markers IL-6 and TNF-α, along with increased levels of the anti-inflammatory marker IL-10, in comparison to those who consumed a carbohydrate drink with equivalent energy and volume.
Galena et al. [26] 2022	Randomized controlled study	The aim of this study was to investigate the practicality of incorporating fermented vegetables into the regular diets of a group of adult women residing in the northeastern region of Florida, United States, and to assess how this dietary change affects markers of inflammation and the composition of gut bacteria.	In this study, various parameters were assessed, including indicators of inflammation, clinical parameters such as the percentage of body fat and systolic blood pressure, and the structure of the gut microbiome, which was evaluated using observed OTUs (Operational Taxonomic Units) and the Shannon index.	34 participants age ranged from 18 to 69 years	United States of America	The results suggest that incorporating approximately 100 grams of fermented vegetables into daily dietary habits over six weeks is both practical and potentially associated with positive alterations in the gut microbiota.
Jung et al. [27] 2023	Randomized controlled trial	The research delves into the possible advantages of Q-CAN® for reducing inflammation and oxidative stress in individuals at risk of cardiovascular issues.	This study assessed various parameters, including indicators of inflammation and oxidative stress, along with blood lipid levels. To be more specific, the research examined the concentrations of high-sensitivity C-reactive protein (hs-CRP), interleukin-6 (IL-6), tumor necrosis factor-alpha (TNF-α), oxidized low-density lipoprotein (oxLDL), and total antioxidant capacity (TAC).	27 participants aged 29 to 75 years	United States of America	The research discovered that the addition of Q-CAN® to the diet did not lead to significant alterations in markers of inflammation or oxidative stress when compared to the control group.
Le Sayec et al. [28] 2022	Randomized controlled trial	The study aimed to examine how supplementing with Aronia berry (poly)phenols influenced artery function and the composition of the gut microbiome in individuals of middle age, both men and women.	The study assessed various parameters, including peripheral office blood pressure (BP), flow-mediated dilation (FMD), pulse-wave velocity (PWV), and augmentation index (AIx). Additionally, blood and fecal samples were collected for analysis. These measurements were taken at three different points: initially at baseline (0 hours), then at 2 hours after the acute consumption of one capsule during visits 1 and 2. These comparisons were made between the Aronia treatment group and the placebo group.	102 participants aged 56.2 ± 9	England	The research revealed that supplementing with Aronia berries enhanced artery function and boosted the richness and diversity of the gut microbiome in middle-aged individuals, including both men and women.
Tingö et al. [29] 2022	Randomized controlled trial	The primary objective was to examine how dual supplementation impacted high-sensitivity C-reactive protein (hs-CRP) levels, while secondary objectives encompassed assessing the influence of the study products on various factors such as anti-inflammatory and pro-inflammatory cytokines, barrier function, osteoarthritis, glucose and insulin levels, the omega-3/omega-6 ratio in the blood, and intestinal fatty acid binding protein (I-FABP) levels.	The study assessed a range of parameters, which included the impact of dual supplementation on hs-CRP levels as well as the influence on anti-inflammatory and pro-inflammatory cytokines, barrier function, osteoarthritis, glucose and insulin levels, the omega-3/omega-6 ratio in the blood, and levels of intestinal fatty acid binding protein (I-FABP). Additionally, exploratory aspects of the study encompassed conducting a Food Frequency Questionnaire (FFQ), measuring vitamin D blood levels, and analyzing short-chain fatty acids (SCFA) in fecal samples.	76 participants aged 65-80 years	Sweden	The eight-week dual supplementation, as explored in this study, did not yield significant effects on the primary measure of hs-CRP. However, it revealed some minor variances in the anti-inflammatory marker IL-10 and the levels of valeric acid in stool samples following the intervention.
van den Belt et al. [30] 2022	Randomized controlled design	This study aimed to examine how Kori-tofu protein impacts blood lipid profiles and various risk factors associated with cardiovascular disease in healthy adults with slightly elevated cholesterol levels.	The study assessed multiple parameters related to lipid and glucose metabolism, which encompassed total cholesterol, low-density lipoprotein (LDL) cholesterol, high-density lipoprotein (HDL) cholesterol, triglycerides, plasma insulin, hemoglobin A1c (HbA1c), fructosamine, and glucose levels. Additionally, blood pressure was measured as an indicator of cardiovascular disease risk.	48 participants aged 40–70 years	Netherlands	The study did not provide conclusive evidence to assert that Kori-tofu yields advantageous impacts on cardiometabolic well-being. Nevertheless, favorable effects on cardiometabolic health were noted in individuals with compromised cardiometabolic profiles, particularly the alterations in measurements related to glycemic control, which hold particular interest in this context.

Discussion

In an era marked by increasingly sedentary lifestyles, dietary habits have taken center stage in discussions of overall health and well-being. In addition to their roles in sustaining energy levels and supporting growth, nutrients play a crucial role in modulating the immune system [31]. The efficiency of the immune system is inherently linked to the body's metabolic state, making it imperative to dissect the nuanced connections between nutrition, metabolism, and immunity.

Cardiovascular Implications

The cardiovascular system, with its intricate network of vessels and organs, is a key player in the regulation of the immune response. Emerging evidence suggests that personalized nutritional interventions can influence cardiovascular health and subsequently affect immune function [32]. In a crossover trial, Basu et al. measured a diverse array of serum metabolites, including both targeted and untargeted compounds, at multiple time points throughout the study. The targeted metabolites included serum branched-chain amino acids (BCAAs), whereas the untargeted metabolites included serum phosphate, benzoic acid, and hydroxyphenyl propionic acid. While only 33 participants with an average age of 53 ± 13 years enrolled in the study, the conclusion suggests that incorporating whole strawberries into one's regular diet may be a beneficial and practical approach to improving the cardiometabolic health of adults [21]. The findings support the notion that strawberry supplementation can positively affect serum metabolic profiles associated with reduced risks of insulin resistance and diabetes as well as endothelial dysfunction in individuals with metabolic syndrome. The notable decreases in targeted metabolites, such as serum BCAAs, and the significant increases in untargeted metabolites, including serum phosphate, benzoic acid, and hydroxyphenyl propionic acid, indicate underlying pathways that contribute to decreasing the risks of cardiometabolic dysfunction [21].

The study conducted by Jung et al. initially enrolled 29 participants, but only 27 adults with at least two risk factors for cardiovascular disease completed the study. Among them, 21 were females and six were males, ranging from 29 to 75 years. This randomized controlled trial aimed to investigate the potential benefits of Q-CAN®, a fermented soy powder, on inflammation and oxidation in individuals with cardiovascular risk [27]. The study measured biomarkers of inflammation and oxidation, such as high-sensitivity C-reactive protein (hs-CRP), interleukin-6 (IL-6), tumor necrosis factor-alpha (TNF-α), oxidized low-density lipoprotein (ox-LDL), and total antioxidant capacity (TAC) [27]. The results showed that Q-CAN® supplementation did not significantly affect the biomarkers of inflammation or oxidation compared to the control group. However, some exploratory findings have indicated potential benefits for specific subgroups of participants. The authors suggest that further research is necessary to fully comprehend the effects of Q-CAN® on these outcomes.

A crossover trial conducted by van de Belt et al. aimed to measure various blood levels, such as total cholesterol, HDL cholesterol, triglycerides, plasma insulin, HbA1c, fructosamine, and glucose in 48 randomly selected participants. Out of these, 45 individuals (including 19 males and 26 females) completed the study, with an average age of 59 years and a BMI of 25.4 kg/m^2^ [30]. This research focused on examining the impact of Kori-tofu protein on blood lipid profiles and other cardiovascular disease risk factors in healthy adults with slightly elevated cholesterol levels. Participants were given a four-week intervention with either Kori-tofu or whey protein, followed by a four-week washout period [30]. Although clear evidence supporting the beneficial effects of Kori-tofu on cardiometabolic health was not found, some positive effects were observed, particularly in those with an impaired cardiometabolic profile. Changes in glycemic control measures were noteworthy in this regard and piqued the potential for future research exploration.

Gastrointestinal Dynamics

The gastrointestinal tract, often referred to as the "second brain," is integral to nutrient absorption and houses a significant portion of the body's immune cells [33]. Understanding the impact of personalized nutrition on gastrointestinal health is paramount to unraveling the complexities of immune regulation.

The Farsi et al. crossover trial consisted of two study phases, each lasting two weeks, separated by a four-week washout period. During the study phases, participants added either 240 g/day of red and processed meat (meat) or mycoprotein-based foods (mycoprotein) to their usual diet. The study revealed that replacing a high intake of red and processed meat with mycoprotein can decrease fecal genotoxicity and nitroso-compounds and increase the abundance of beneficial microbes such as *Lactobacilli*, *Roseburia*, and *Akkermansia* as well as the excretion of short-chain fatty acids in the gut [24].

Galema et al. investigated the viability of consuming fermented vegetables regularly and its effects on inflammation markers and gut bacteria profiles in a convenience sample of adult women living in the northeast region of Florida, United States. They determined the feasibility by assessing compliance with the interventions (groups A and B) and participants' reports on their tolerance to daily vegetable consumption. Compliance was measured using two factors: the number of days in the study and the total amount of vegetables consumed. If the interval between the first and second study visits was within 42 ± 3 days, compliance with the study duration was considered 100% [26]. Regarding the results, out of 85 potential participants who were screened before the eligibility assessment, 34 participants were randomly assigned to one of the three treatment groups, and 31 completed the study. The most common reason for exclusion from the study was the presence of autoimmune diseases or other chronic illnesses such as diabetes or heart disease. All study objectives were evaluated by at least nine participants from each group. Several baseline characteristics were balanced between the groups, except for age and dietary variables. Participants in the control group (group C) were younger than those in the other two groups [26].

Differences in calorie intake, macronutrients, and fiber were evident among the three groups. However, no significant differences were observed for any of the parameters examined. Pairwise comparisons within each group revealed that the control group (group C) had a significantly lower percentage of body fat and systolic blood pressure at the end of the study. These changes were accompanied by significant decreases in the total calorie and macronutrient intake in this group. The results of this pilot and feasibility study suggest that it is feasible for Western females to consume 0.5 cups of fermented cabbage and/or cucumbers every day for six weeks, with the caveat that some individuals may experience common side effects such as bloating [26].

According to a study conducted by Le Seyac et al., supplementation with Aronia berries improved arterial function and increased the richness and diversity of the gut microbiome in middle-aged men and women [28]. These results suggest that Aronia berries may provide cardioprotective benefits and could be a useful dietary intervention for improving cardiovascular health. The study measured several outcomes, including peripheral office blood pressure, flow-mediated dilation, pulse-wave velocity, and augmentation index, as well as blood and fecal samples [28]. These measurements were taken at baseline and two hours post-consumption of one capsule on visits 1 and 2, as well as at 12 weeks. The study included 323 volunteers, with 221 excluded and 102 (47 men, 55 women) randomly assigned to the Aronia or control groups as the intention-to-treat (ITT) sample. Ninety-seven participants completed all visits, with a 5% dropout rate, and were included in the acute, chronic, and acute-on-chronic analyses [28].

Metabolic Crossroads

Metabolism serves as a biochemical engine driving cellular processes, and its influence extends to immune functions [34]. The objective of Abuawad et al.'s study was to explore the effects of folic acid (FA) and creatine supplementation on arsenic methylation indices and metabolite concentrations in a Bangladeshi population. In total, 622 individuals were enrolled in this study. Six participants dropped out due to adverse effects, three due to pregnancy, two withdrew from the trial, and one was excluded from the analysis because of missing blood samples. Consequently, 609 participants completed the study and were included in the final analysis. This study measured the concentrations of arsenic (As) metabolites, including the primary methylation index (PMI, calculated as monomethyl arsenic [MMA]/inorganic As) and the secondary methylation index (SMI, calculated as dimethyl arsenic species [DMAs]/MMAs) in the blood of Bangladeshi adults with varying folate levels. Additionally, the study examined the potential reversal of the treatment effects on As metabolites following the discontinuation of FA treatment during phase 2 (weeks 12-24) [18]. Supplementation with FA resulted in decreased levels of blood MMAs (bMMAs) and increased levels of blood DMAs (bDMAs) among a group of mostly folate-sufficient adults, whereas creatine supplementation led to a reduction in bMMAs. The reversal of these effects after discontinuing FA supplementation indicates its short-term benefits, emphasizing the need for long-term interventions, such as fortifying FA [18].

Arbizu et al. assessed numerous anthropometric and physiological markers, including body weight, height, BMI, waist and hip circumference, body temperature, systolic and diastolic blood pressure, heart rate, and oxygen saturation, as well as blood samples obtained from 60 adult participants aged 18 years or older to analyze lipid profiles, glucose levels, and liver enzymes. This study also evaluated dietary intake and adherence to the Dietary Guidelines for Americans (DGA) using the Healthy Eating Index (HEI). The objective of the study was to investigate the effects of dark sweet cherry supplementation on blood pressure and inflammation in obese adults, and the results showed that supplementation with dark sweet cherry reduced blood pressure and pro-inflammatory interferon-gamma (IFNγ) in obese adults without affecting lipid profile, glucose levels, and liver enzymes [20]. There were no notable differences in anthropometric and physiological measurements between the cherry and placebo groups, except for a significant decrease in systolic blood pressure (SBP) in the cherry group compared with the placebo group. Furthermore, the study found that individuals with high BMI experienced a significant decrease in ∆ SBP compared to the placebo group due to the dark sweet cherry intervention [20].

Ben-Yacov et al. examined the impact of the gut microbiome on diet and human health, specifically focusing on the effects of personalized postprandial-targeting diets on cardiometabolic markers in pre-diabetic individuals. This study assessed various cardiometabolic markers, such as glucose levels, insulin resistance, lipid levels, and body weight, among others. Additionally, the researchers conducted a comprehensive analysis of the six-month dietary and microbiome compositional changes observed in the 200 participants who completed the intervention. Of the 800 participants screened for eligibility, 688 were randomly assigned to the intervention or control group. The study's findings suggest that personalized nutrition based on an individual's gut microbiome may have implications for the development of personalized dietary interventions for other health conditions beyond pre-diabetes [22]. The researchers identified nine microbial species that partially mediate the association between specific dietary changes and clinical outcomes, including three species from *Bacteroidales*, *Lachnospiraceae*, and *Oscillospirales *orders that mediate the association between postprandial-targeting (PPT)-adherence score and clinical outcomes of hemoglobin A1c (HbA1c), high-density lipoprotein cholesterol (HDL-C), and triglycerides [22]. This may serve as a foundation for future mechanistic experiments on their role in human diet and health as well as potential therapeutic directions to be evaluated in preclinical and intervention studies to improve cardiometabolic health in pre-diabetes. The researchers also used machine-learning models trained on dietary changes and baseline clinical data to predict personalized metabolic responses to dietary modifications and assess the importance of features for clinical improvement in cardiometabolic markers of blood lipids, glycemic control, and body weight [22].

Inflammatory Interfaces

Inflammation, the cornerstone of the immune response, shows intricate connections with nutrition and metabolism. The interplay between pro- and anti-inflammatory dietary components shapes the immune landscape [35]. The study conducted by De Giuseppe et al. evaluated the impact of *Camelina *oil-enriched crackers on serum omega-3 levels, inflammatory markers, and lipid profiles in adults aged 65 and above. Of the initial 66 participants, 50 completed the study because of attrition and COVID-19 restrictions. The participants were randomly assigned to either the experimental group (n = 34) or the control group (n = 32) in a 1:1 ratio [23]. The primary outcomes measured included serum levels of eicosapentaenoic acid (EPA), docosahexaenoic acid (DHA), linoleic acid (LA), and arachidonic acid (ARA). Secondary outcomes assessed included inflammatory markers, such as pro-inflammatory cytokines (IL-18 and TNF-alpha), anti-inflammatory cytokines (TGF-beta 1 and TGF-beta 2), and CRP levels, as well as lipid panel parameters, including total cholesterol (TC), high-density lipoprotein cholesterol (HDL), low-density lipoprotein cholesterol (LDL), and triglyceride (TG) concentrations. The results showed that *Camelina* oil-enriched crackers significantly increased serum omega-3 levels as evidenced by a higher alpha-linolenic acid concentration in the camelina group compared to the placebo group (p < 0.01) [23]. However, no significant changes were observed in the inflammatory markers and lipid profiles. The study suggests that *Camelina *oil could be a sustainable and healthy alternative to traditional oils for improving the nutritional status and health of older adults [23].

Fraschetti et al. investigated the immediate consequences of milk intake on systemic inflammation following a combination of resistance and plyometric exercise in young adult females. This study explored the potential benefits of milk consumption in reducing post-exercise inflammation. The study measured the concentrations of inflammatory biomarkers (IL-6, TNF-α, and IL-10) in the blood of untrained females who consumed either skim milk or an isoenergetic, isovolumetric carbohydrate beverage following high-intensity/impact exercise [25]. The study included 13 females with an average age of 20.3 ± 2.3 years. The results indicated that consuming skim milk after high-intensity/impact exercise led to lower levels of the inflammatory biomarkers IL-6 and TNF-α and higher levels of the anti-inflammatory biomarker IL-10 compared to consuming an isoenergetic, isovolumetric carbohydrate drink [25].

A study conducted by Tingö et al. aimed to evaluate the effectiveness of combining probiotics and omega-3 supplements in reducing inflammation among older adults, specifically those aged 65-80 years. A total of 76 participants were enrolled, with 39 in the control group and 37 in the intervention group. The eight-week supplementation regimen showed no significant impact on the primary outcome measure, hs-CRP but revealed some minor differences in the anti-inflammatory marker interleukin (IL)-10 and stool levels of valeric acid [29]. Overall, the results suggest that the combination of probiotics and omega-3 supplements has moderate anti-inflammatory effects and could potentially be used as a non-pharmacological treatment for low-grade inflammation in the elderly. Further research is required to determine the potential benefits of these supplements in managing inflammation in older adults as suggested by the study authors [29].

Limitations

The study faces a significant challenge in exploring the wide range of dietary interventions available, from diverse nutritional strategies to various supplementation regimens. This complexity necessitates careful consideration and comprehensive analysis of their respective impacts on metabolism and immunity. However, capturing the full spectrum of dietary interventions and assessing their influence on health is difficult, given the numerous dietary variables that can influence the outcomes. Additionally, the study grapples with the complexity of assessing wellness, inflammation, and health using multiple markers, from biochemical markers to clinical assessments. Harmonizing these diverse indicators to form a cohesive understanding of the intervention's effects is a methodological challenge that raises concerns about selective reporting bias and underscores the need for a nuanced interpretation of the study's findings in the context of health and immune function.

The temporal scope of the study presents limitations due to its reliance on recent literature, potentially excluding valuable contributions from earlier research and limiting the synthesis of available knowledge. To ensure a comprehensive understanding, a balance between current evidence and historical context is necessary. Caution should be exercised when extrapolating the results to diverse demographic groups. Additionally, the study's participant demographics, consisting predominantly of White individuals from the United States, raise concerns about the generalizability of the findings to more diverse populations as dietary responses and immune interactions can vary across ethnic and cultural groups. The study's short-term follow-up design restricts the evaluation of long-term effects on academic achievement. While it offers valuable insights, the limited time frame highlights the need for a more extended follow-up to assess the intervention's durable impacts on educational outcomes.

## Conclusions

The study concludes that personalized nutrition can play a significant role in optimizing immune health by addressing specific nutrient needs and supporting immune function. Emphasis must be placed on the importance of being a good patient and taking an active role in one's health care. The RCTs utilized in this study covered various nutritional parameters, which are representative of society's consumption of different nutrients, and it was shown to have a positive correlation to a vast array of health benefits. By asking questions, being an advocate for oneself, and building good relationships with healthcare providers, patients can improve their outcomes and achieve better health. It is important to remember that healthcare is a partnership between patients and providers and that each party plays a role in ensuring the best possible care. By following these tips and being proactive in one's healthcare, patients can achieve better health and quality of life. However, it is essential to acknowledge that this field continues to evolve. While preliminary studies have shown encouraging results, further research is needed to establish the effectiveness of personalized nutrition in enhancing immune responses and reducing the risk of infectious diseases.
